# Old and New Players of Inflammation and Their Relationship With Cancer Development

**DOI:** 10.3389/fonc.2021.722999

**Published:** 2021-11-22

**Authors:** Rodolfo Chavez-Dominguez, Mario Perez-Medina, Dolores Aguilar-Cazares, Miriam Galicia-Velasco, Manuel Meneses-Flores, Lorenzo Islas-Vazquez, Angel Camarena, Jose S. Lopez-Gonzalez

**Affiliations:** ^1^ Departamento de Enfermedades Cronico-Degenerativas, Instituto Nacional de Enfermedades Respiratorias “Ismael Cosio Villegas”, Mexico City, Mexico; ^2^ Posgrado en Ciencias Biologicas, Universidad Nacional Autonoma de Mexico, Mexico City, Mexico; ^3^ Laboratorio de Quimioterapia Experimental, Departamento de Bioquímica, Escuela Nacional de Ciencias Biológicas, Instituto Politécnico Nacional, Mexico City, Mexico; ^4^ Departamento de Patología, Instituto Nacional de Enfermedades Respiratorias “Ismael Cosio Villegas”, Mexico City, Mexico; ^5^ Laboratorio de Human Leukocyte Antigen (HLA), Instituto Nacional de Enfermedades Respiratorias “Ismael Cosio Villegas”, Mexico City, Mexico

**Keywords:** inflammation, chronic inflammation, cancer development, cancer-related inflammation, innate immune response, adaptive immune response, cancer immunoediting theory, immune checkpoint molecules

## Abstract

Pathogens or genotoxic agents continuously affect the human body. Acute inflammatory reaction induced by a non-sterile or sterile environment is triggered for the efficient elimination of insults that caused the damage. According to the insult, pathogen-associated molecular patterns, damage-associated molecular patterns, and homeostasis-altering molecular processes are released to facilitate the arrival of tissue resident and circulating cells to the injured zone to promote harmful agent elimination and tissue regeneration. However, when inflammation is maintained, a chronic phenomenon is induced, in which phagocytic cells release toxic molecules damaging the harmful agent and the surrounding healthy tissues, thereby inducing DNA lesions. In this regard, chronic inflammation has been recognized as a risk factor of cancer development by increasing the genomic instability of transformed cells and by creating an environment containing proliferation signals. Based on the cancer immunoediting concept, a rigorous and regulated inflammation process triggers participation of innate and adaptive immune responses for efficient elimination of transformed cells. When immune response does not eliminate all transformed cells, an equilibrium phase is induced. Therefore, excessive inflammation amplifies local damage caused by the continuous arrival of inflammatory/immune cells. To regulate the overstimulation of inflammatory/immune cells, a network of mechanisms that inhibit or block the cell overactivity must be activated. Transformed cells may take advantage of this process to proliferate and gradually grow until they become preponderant over the immune cells, preserving, increasing, or creating a microenvironment to evade the host immune response. In this microenvironment, tumor cells resist the attack of the effector immune cells or instruct them to sustain tumor growth and development until its clinical consequences. With tumor development, evolving, complex, and overlapping microenvironments are arising. Therefore, a deeper knowledge of cytokine, immune, and tumor cell interactions and their role in the intricated process will impact the combination of current or forthcoming therapies.

## Introduction

In the human body, cells in the organs or tissues are continually exposed to pathogenic infections or distinct genotoxic insults that damage the host cells. The host’s immune system triggers an inflammatory reaction in response to recognition of diverse molecules released by the pathogens and damaged tissues. This dynamic process in time and space requires a strict coordination and regulation of cellular and molecular events to delimit and eliminate damage-causing agents. It also involves repair of damaged tissues to restore the typical tissue architecture, thus maintaining homeostasis. Chronic inflammation occurs when mechanisms involved in the activation or regulation of inflammation are dysregulated. This persistent inflammatory state has been associated with distinct pathologies, such as obesity, metabolic disorder, allergies, autoimmune diseases, and most importantly the risk for cancer development. Since the nineteenth century, the relationship between inflammation and cancer has been well known, and currently, approximately 25% of cancers arise from a chronic inflammatory condition that could be elicited under sterile or non-sterile environments. This chronic inflammation causes the incessant recruitment of several immune cells, which are implicated in the production and release of genotoxic agents for cell transformation. Importantly, oncogenic changes promote activation of inflammatory pathways in malignant cells to release molecules that perpetuate and strengthen the inflammatory phase of chronic inflammation. In the microenvironment, continuous production and release of cytokines, chemokines, and growth factors sustain tumor growth and its survival.

This review highlights classic and new players participating in complex and redundant interactions, which trigger signaling pathways involved in the acute inflammatory process and wound healing resolution as a homeostatic process. Some events that deregulate and amplify an inflammatory reaction resulting in a chronic inflammation are also revised. During this persistent stage, several environmental factors might be involved in the development of a nascent tumor based on the cancer immunoediting concept that implicates the role of the inflammatory immune response in tumor development. Therefore, this review aimed to depict some immunologic events that participate in the recognition and elimination of nascent tumor cells during the spatial and temporal processes. In case of failure to eradicate some of the transformed cells by the immune cells or gradual occurrence of new tumor cell clones, resisting the impact of cytocidal immune cells, some cellular processes leading to a second phase known as equilibrium are described. In the tumor mass, new clones harboring more genetic alterations become preponderant that increase the tumor heterogeneity. This increased clonal diversity leads to the acquisition of novel resistance mechanisms to evade the cytocidal arsenal of effector immune cells. In addition, tumor cells could generate a tumor microenvironment that gradually shift the phenotype of the tumor-infiltrating immune cells to sustain tumor growth until clinical implications. In brief, we indicate the role of inflammation through the concept of cancer immunoediting, and denote the plasticity of immune cells to antagonize or promote tumor growth from cell transformation to tumor progression. Finally, the use of current and novel anti-inflammatory drugs in the prevention and treatment of cancer will be discussed

## Acute Inflammation

Inflammation is a self-protective response against the presence of distinct cellular harmful agents, i.e., exogenous or endogenous. In this setting, inflammation could be elicited in non-sterile or sterile environments caused by infectious organisms or toxins, external bodies, chemicals, dead cells, tissue damage, and endogenous metabolites (1). In the case of pathogen infection, pathogen-specific molecules, by-products of bacterial degradation, or metabolism act as pathogen-associated molecular patterns (PAMPs). In addition, stressed cells injured by the pathogen’s own metabolism translocate internal proteins into the cell membrane or release intracellular molecules. Furthermore, fragments from extracellular matrix components are released by proteases during cell death, a process dependent or independent of the infection. These damage-associated molecular patterns (DAMPs), together with PAMPs, alert the immune cells from the damage (2, 3). In addition to PAMPs and DAMPs, homeostasis-altering molecular processes (HAMPs) are emerging as new players in inflammation and currently encompass various endogenous small lipophilic metabolites, such as lysophospholipids that regulate cellular homeostasis at physiological concentrations. However, in the presence of sterile or non-sterile agents causing cellular stress, the HAMP concentration is modified and sensed by intracellular molecules, thereby triggering inflammation (4).

Independent on the source, immune and non-immune cells recognize PAMPs, DAMPs (1) and HAMPs through distinct receptors. PAMPs and DAMPs are recognized through membrane molecules known as pattern recognition receptors (PRRs), whereas HAMPs are sensed by nuclear receptors such as the thyroid hormone receptor, vitamin D receptor, estrogen receptor (ER), androgen receptor, glucocorticoid receptor, and PR, as well as adopted orphan receptors such as farnesoid X receptor (FXR), RAR-related orphan receptor (ROR), and PPARs (5).

Once PAMPs and DAMPs are released, these molecules impact the endothelial cells, triggering vascular dilation, enhancing capillary permeability, decreasing tight junction integrity and blood flow. With regard to HAMPs, some of them regulate cellular functions within the cytoplasm. On endothelial cells, estrogens bind to the corresponding ER in the cytoplasm to activate signaling pathways controlling the vascular tone and endothelial cell migration (6).

In an initial step, increased expression of adhesion molecules, such as ICAM and PECAM1, in the blood vessels induce platelet cell aggregation causing vasoconstriction and blood clots to reduce blood loss. The uninterrupted blood vessel dilation induces expression of more adhesion molecules, increasing platelet adherence to the endothelium. In addition, activated platelets secrete several growth factors such as epidermal growth factor (EGF) or platelet-derived growth factor (PDGF), histamine, serotonin, and von Willebrand factor for clot stabilization. Furthermore, platelet degranulation activates the complement cascade releasing anaphylatoxins to support neutrophil transmigration. Likewise, activation of coagulation cascade also releases vasoactive mediators such as fibrinogen and fibronectin. This local microenvironment facilitates the leukocyte attachment to initiate their extravasation to the injured zone (7, 8). Additionally, local increase of PAMPs and DAMPs serves as “find me” signals for attracting tissue and blood cells, as they are recognized as “danger signals”. Neutrophils, dendritic cells (DC), monocytes, and other immune cells must be recruited from the circulating blood to the injured site. Transmigration through the endothelial cell wall by these cells is supported by continuous expression of distinct classes of adhesion molecules belonging to the immunoglobulin superfamily such as integrins.

As mentioned above, PAMPs and DAMPs are recognized by the immune cells using distinct types of membrane and cytoplasmic PRRs. Based on their localization, PRRs are classified into membrane-bound receptors such as Toll-like receptors (TLRs), C-type lectin receptors, and cytoplasmic receptors, such as the nucleotide-binding domain leucine-rich repeat receptors (NLRs), retinoic acid-inducible gene-I (RIG-I)-like receptors (RLRs), absent in melanoma-2 (AIM-2)-like receptors (ALRs), and protein-containing tripartite motif and receptor for advanced glycation end-products (RAGE) (9). PAMP and DAMP molecules bind to TLRs and NLR, stimulating the activation of several signaling pathways involved in a cascade of multi-protein complexes such as the inflammasome consisting of NLR, ASC adaptor protein, and pro-caspase 1 (10). Recent evidence suggests that the inflammasome component NOD-, LRR-, and pyrin domain-containing 3 (NLRP3), in addition to the direct interaction with PAMPs and DAMPs, also detect HAMPs, thereby modulating the inflammatory response (4, 5, 11).

Activation of inflammasome leads to caspase-1-mediated cleavage of proinflammatory cytokines interleukin (IL)-1 and IL-18 into their active form. In addition, interaction of PAMPs or DAMPs with TLRs can activate intracellular molecules, such as the transcription factor nuclear factor-κ B (NF-κB) and mitogen-activated protein kinases pathway. These pathways control the expression of many genes to synthesize proinflammatory lipids, cytokines, and chemokines such as monocyte chemoattractant protein-1 (MCP-1), tumor necrosis factor α (TNF-α), IL-6, IL-8, and IL-23 for maintaining and perpetuating the inflammatory response (12). Ishikawa et al. reported that the cyclic GMP-AMP synthase (cGAS) and stimulator of interferon genes (STING) pathways trigger inflammation associated with pathogen infection. In this setting, the sensor cGAS recognizes cytoplasmic DNA, acting as danger signal, and stimulates the production of second messenger cyclic GMP-AMP, which activates STING. This pathway leads to NF-kB activation, triggering a type I interferon-dependent inflammatory reaction (13, 14). Additionally, nuclear receptors have been described to also modulate the synthesis of cyclic nucleotides, such as cAMP and cGMP (15). However, further rigorous studies on this proposal are required.

This altered homeostatic environment attracts polymorphonuclear neutrophils. Neutrophils are the most abundant white blood cells in the circulation and are considered as the first line of defense of the immune system. They are rapidly recruited to damaged sites where they phagocyte pathogens and undergo degranulation. Neutrophil cytotoxic granules contain enzymes with antimicrobial activity such as defensins, cathelicidins, myeloperoxidase, lactoferrins, and cathepsins. In addition, the release of their nuclear content generates a meshwork of chromatin and protease extracellular fibers known as neutrophil extracellular traps (NET) (16). In ischemia/reperfusion damage of the liver, release of NETs is rather mediated by binding of DAMPs, such as HMGB1 and histones, to TLR-4 or TLR-9. In addition to NETs, regulated necrotic cell death such as pyroptosis, necroptosis, and ferroptosis stimulates the production and release of some pro-inflammatory cytokines such as IL-1 β, IL-6, TNF-α, MCP-1, and CXCL-10, thereby propagating the inflammatory environment (17–19).

Moreover, the synthesis and release of reactive oxygen and nitrogen species (RNOs) such as superoxide, hydrogen peroxide (H_2_O_2_), hydroxyl radical, nitric oxide, peroxynitrite, and hydrochlorous acid is enhanced by the oxidative burst, causing collateral oxidative damage in the harmful agent and surrounding tissues (20). Other studies indicate that the release of lysosomal content from neutrophils is required to induce the inflammasome activation (21, 22). Their phagocytic and microbicidal activities are crucial to prevent the spread of microorganisms, facilitate cell death, and limit the tissue damage by maintaining a local concentration of enzymatic molecules.

Subsequently, tissue macrophages and mainly blood monocytes are recruited in the damaged site, differentiating into mature macrophages whose main function is the phagocytosis of microbes, cellular debris, and dead cells (23). The continuous migration of monocytes and other immune cells is sustained by the local production of several proinflammatory lipid mediators derived from arachidonic acid, CXC- and CCL-chemokines, and proinflammatory cytokines, such as, IL-1α, IL-1β, TNF-α, IL-6, etc. (24, 25). Besides the recruitment of these cells, particular plasmatic proteins are activated such as the complement system, which promotes destruction and opsonization of microbial agents *via* the lectin pathway, enhancing the recruitment of immune cells (23). In addition, oxidized lipids from dead cells are recognized and presented as non-self-antigens because they are recognized as dangerous biological waste of the host. These oxidation-specific components are recognized as endogenous DAMPs by PRR in phagocytic cells acting as antigen-presenting cells (APCs) to over-stimulate the innate immune cells (26). Recent evidence reported that CD1b in dendritic cells play a role in oxidizing lipids that stimulate NKT cells (27). Leiw et al. reported that self-antigenic lipids are associated with CD1d that promotes NKT cell participation in restoring tissue homeostasis after a sterile injury (28).

Collectively, all soluble factors released by neutrophils, macrophages, DCs, and stromal cells, such as fibroblast, mast, and endothelial cells, regulate the amplitude and duration of the inflammatory response acting as a self-amplifying network.

Clearance of foreign pathogens, cell debris, and dead cells promotes resolution of inflammation in a harmonious and active process. Recent evidence showed that inflammation resolution proceeds in synchronic and overlapping phases. The whole process also includes cessation of neutrophil tissue infiltration, regulation in cytokine-chemokine production, elimination of death neutrophils and their immediate efferocytosis mediated by macrophages, return of viable cells into the blood or lymphatic system, successful outcome of the wound healing response, and new tissue formation for homeostasis restoration (29).

Inflammation resolution is managed by the production of an array of molecules with anti-inflammatory and immunomodulatory activities called pro-resolving mediators. Some of these molecules are derived from the catabolism of synthesized lipids during the acute inflammatory phase. For example, arachidonic and eicosapentanoic acid promote lipoxin and prostaglandin production, whereas docosahexanoic acid promote maresin, resolvin, and protectin release (29–31). These lipid mediators are produced by recruited neutrophils and macrophages, as well as endothelial cells, epithelial cells, and platelets through the lipoxygenase enzyme. In addition to lipid mediators, proteins such as Annexin-A1 show a potent anti-inflammatory and pro-resolving activities. Most of these pro-resolving mediators exert their function by binding to a wide array of G-protein coupled receptors (GPCR) activating diverse pathways to produce immunoregulatory molecules (29). Recent reports by Wang et al. revealed that lysophosphatidylserine, acting as a HAMP, might act as a pro-resolving mediator because it binds to GPCR 34, which plays a role in anti-inflammatory responses (32). Additionally, pro-resolving mediators influence the rest of the steps involved in inflammation resolution.

Neutrophil recruitment to the damaged site ceases when the stimuli triggering the inflammation disappeared, leading to endothelial inactivation by decreased expression of cell adhesion molecules and reduced vasodilation. In this way, Annexin-A1 and/or its analog peptides play a crucial role as a stop signal for neutrophil extravasation. Evidence showed that Annexin-A1 or its mimetic peptides decreased the production of proinflammatory cytokines such as IL-1 β, IL-8 and CXCL1 and the expression of VCAM-1, ICAM-1, and E selectin adhesion molecules, thereby inhibiting the capture of circulating neutrophils on the activated endothelium (33, 34). Another way to limit the infiltration of neutrophils to the inflammation site is by dismantling the established chemokine-cytokine gradients. In this setting, aggregated NETs promote IL-8 and IL-1 β degradation, mediated by serine proteases that are released by neutrophils and macrophages (35).

Additionally, clearance of recruited neutrophils is controlled by the induction of regulated non-necrotic cell death (19). In an acute inflammation, the lifespan of neutrophils is enhanced by the release of proinflammatory cytokines, growth factors such as granulocyte-monocyte colony-stimulating factor (GM-CSF), and microbial derived products. However, through the resolution phase of inflammation, the lifespan of neutrophils is reduced by macrophages, inducing neutrophil death through the release of agonistic molecules for death receptors such as Fas ligand (FasL), TNF-α, and TNF-related apoptosis-inducing ligand (TRAIL) (36). Recent evidence demonstrated that IFN-β is also important to induce inflammatory neutrophil death by activating STAT3 during a non-sterile inflammation caused by *Escherichia coli* (37). Dead neutrophils are engulfed by macrophages in a process called efferocytosis. During efferocytosis, phosphatidylserine exposed on the cell surface of dying neutrophils or apoptotic bodies acts as an “eat me” signal, activating distinct intracellular pathways for reprogramming of inflammatory M1 into anti-inflammatory and pro-resolving M2 macrophages (38). Kourtzelis et al. demonstrated that the release of developmental endothelial locus-1 promotes efferocytosis of death neutrophils by interacting with exposed phosphatidylserine on dying cells and α_v_β_3_ integrin receptors on macrophages in a mouse model of periodontitis (39). In addition, type I interferons are crucial to promote reprogramming of M1 into M2 macrophages since knockout IFN-β genes in macrophages reduce their ability to release anti-inflammatory cytokines (37). Several studies indicated that M2 macrophages produce IL-10 and transforming growth factor-beta (TGF-β) in addition to PDGF, vascular endothelial growth factor (VEGF), and other mediators. Reprograming of macrophages also impacts the gradual shifting of T-lymphocytes, changing from Th1 to Th2 phenotype, regulatory T-lymphocytes (Treg cells), or other immunoregulatory cell subpopulations (40, 41). Additionally, the recruitment and differentiation of Treg cells are essential for inflammation resolution. Recent evidence showed that Tregs participate in the reduction of atherosclerosis plaques in mice. Depletion of Tregs impairs the resolution phase of inflammation in atherosclerosis causing a perpetuation of the inflammatory reaction and decreasing efferocytosis and production of pro-resolving mediators (42). Moreover, other immune cells such as innate lymphoid cells and myeloid-derived suppressor cells (MDSCs) have been demonstrated to participate in the resolution of inflammation in distinct pathologies (43, 44).

Through the acute phase of inflammation and its resolution process, a myriad of cells is recruited at the damaged site due to the established chemokine-cytokine gradients. The recruitment of monocyte-derived macrophages, platelets, fibroblast, and other cell types is essential for the wound healing process, producing a plethora of wound-related signals. Growth factors, such as PDGF, VEGF, fibroblast growth factor (FGF), keratinocyte growth factor-1, and EGF, and chemokines and cytokines promote the proliferation of distinct sets of cells as a prerequisite for wound healing (45).

As the inflammation subsides, proliferation becomes a major theme with the focus on covering the wound surface, restoring the vascular network, and forming new connective tissues (granulation tissues). This proliferative phase is characterized by angiogenesis, a process essential for restoration of nutrient and oxygen supply. This process requires growth factors such as VEGF, PDGF, basic FGF (bFGF), and thrombin, promoting the proliferation and migration of endothelial cells toward the site of angiogenic stimulus (46). These sprouts develop into endothelial tubules that connect with each other to form the vessel lumen, and these new vessels interact with pericytes and smooth muscle cells forming a network of venules and arteries. In addition, bone-marrow-derived endothelial progenitors also participate in forming *de novo* vessels, a process known as vasculogenesis (46).

Fibroblasts are other cells that play a central role in repairing injured tissues. During this proliferative phase, fibroblasts are recruited to the provisional matrix and proliferate in response to the secreted cytokines and growth factors PDGF, TGF-β and bFGF produced by platelets and macrophages in the wound (45). When the wound condition is maintained, circulating bone-marrow-derived mesenchymal progenitors called myofibroblast migrate to the injured area. These cells secrete chemokines, cytokines, and growth factors that strengthen their local concentration to promote healing. Myofibroblasts, besides enhancing angiogenesis, act as APCs that stimulate immune cell infiltration. Other cells in the tissue, in addition to fibroblast, such as pericytes and epithelial cells, have been reported to differentiate myofibroblast in the uninjured zone (47). In this sense, some reports suggest that subsets of macrophages identified by CD45, CD11b, and F4/80 molecules transit to myofibroblast-producing growth factors such as MCP-1, TGF-β, and VEGF contributing to new blood sprouting during angiogenesis (48).

The remodeling phase is the last process in resolving inflammation. During this reparative phase, recruited fibroblasts produce zinc-dependent endopeptidases known as metalloproteinases to degrade the provisional matrix and produce other ECM components, such as proteoglycans, glycosaminoglycans, fibronectin, hyaluronic acid, and collagen to fill the wound gap. In this phase, wound contraction occurs and participation of the myofibroblast is crucial as they produce α-smooth muscle actin and collagen, as responses to fibronectin and other proteins to ECM. Reports indicated that macrophages shift from a M2a to a M2c profile showing fibrolytic activity, as they release proteases for ECM degradation and engulf excess cells present in the damaged site (49).

In addition, myofibroblasts bind to each other allowing wound healing and are eliminated by cell death once tissue integrity is reached. Collagen I is overproduced to promote greater tensile strength. Finally, the formation of new blood vessels and cellular infiltration is avoided, establishing an acellular milieu during wound closure.

Although in recent years the cellular and molecular mechanisms involved in inflammation resolution have been characterized, several aspects remain relatively unclear, e.g., the whole signals that cause the gradual shift from acute inflammation to the resolution or interaction among cells participating in this process. Exhaustive investigation in crucial points of this phenomenon must be performed in order to have a deeper knowledge of the process.

## Chronic Inflammation

As described above, inflammation is a self-limiting process of restoring tissue homeostasis after a non-sterile or sterile source of damage that causes injury. However, when this process persists during the inflammatory phase and is dysregulated or the body is unable to repair the damaged tissue, inflammation is prolonged and exacerbated leading to further damage of the surrounding healthy tissues. This uncontrolled state, denominated as chronic inflammation, involves a persistent inflammatory stage caused by the noxious stimulus. Chronic inflammation is characterized by abundant neutrophil infiltration and profuse presence of RNOs and tissue-damaging enzymes. All these factors maintain a positive feedback loop perpetuating the inflammatory process and increasing the damage on the surrounding healthy tissues.

Distinct pathological conditions have been associated with chronic inflammation in the host such as chronic disease, diabetes, malnutrition, vascular insufficiency, and aging, among others, and factors as recurrent trauma, tissue necrosis by hypoxia or ischemia, edema, pressure, and infection (50). Some mechanisms underlying the chronic inflammation have been proposed, such as inefficient elimination of damaging agents by the immune cells, alteration in their activity, and dysregulation of cell signaling pathways involved in the resolution phase (50).

The etiology of chronic wounds is diverse, and their causes are not fully understood despite the efforts made to identify them. With regard to non-sterile inflammation, persistent infection constantly releases PAMPs. In the case of intracellular pathogens, DAMPs are released due to continuous injury and cell death. Besides, the perpetual release of some ECM fragments from the damaged tissue exacerbates the local concentration of released DAMPs. When PAMPs or DAMPs are recognized by distinct PRRs, these receptors trigger the synthesis of proinflammatory cytokines. N-formyl peptides, present in the bacterial membrane or released by dying cells, act as potent chemoattractant for platelets and phagocytic cells. In neutrophils, N-formyl peptide signalization induces IL-8 secretion, another molecule with potent chemoattractant activity (51). In addition, cytochrome-c, cardiolipin, succinate, and other DAMPs trigger different signaling pathways with proinflammatory properties that promote and potentiate the inflammatory response. When these events are not orchestrated, they progress to the development of a chronic inflammation (52). A good example of this phenomenon is the release of intracellular nucleotides. In a regulated inflammatory process, injured or dying cells release ATP to alert the immune system. ATP binds to the P2 purinergic receptors, widely expressed in different tissues, contributing to the blood flow regulation and vascular endothelium activation and promoting immune cell phagocytosis (53). However, dysregulated release of ATP leads to chronic inflammation by RNOs overproduction. Tatsushima et al. demonstrated that in a mice model of steatohepatitis, a chronic liver inflammation, the release of vesicular ATP is crucial in promoting inflammation, fibrosis, and macrophage infiltration. In this regard, knockout vesicular nucleotide transporter gene in mice eliminated the damage caused by high fat diet (54).

Recently, a regulated cell death process known as iron-dependent programed cell death has been linked to chronic inflammation. In inflammatory zones showing increased extracellular iron, surrounding cells capture this metallic compound by endocytosis. The iron released in cytosol increases the ROS levels, generate lipid peroxidation with the concomitant cell membrane disruption, phenomenon known as ferroptosis. As consequence of this process, more DAMPs are released to sustain the chronic inflammatory process. As was previously mentioned, oxidized lipids mediators also contribute to chronic inflammation by activating enzymes related to respiratory burst, thus increasing the oxidative metabolism of the cells in the microenvironment (18, 19, 55).

Impaired inflammation resolution leads to aberrant tissue remodeling and organ dysfunction; therefore, constant cell damage triggers the release of endogenous lipids. The cellular and molecular mechanisms leading to pathogenesis of chronic inflammation, in which the bioactive lipids act, has been recently reported by Chiurchiú et al. (56).

Excessive or uncoordinated production of lipids, DAMPs, and/or PAMPs may lead to a dynamic imbalance of intracellular signals, resulting in chronic inflammation. With regard to HAMPs, increased levels of some lysophospholipids such as LPC are associated with the expression of cyclooxygenase type 2 enzyme in the endothelial cells to produce proinflammatory lipids derived from the arachidonic acid (57). This evidence presented thus far supports the role of uncontrolled production and release of PAMPs, DAMPs, or HAMPs as an event promoting chronic inflammation in malignant and non-malignant diseases.

As discussed earlier, excessive neutrophil and macrophage infiltrations are considered a crucial factor involved in chronic inflammation. Neutrophil accumulation results in RNOs overproduction and protease release, which damages the ECM, as well as the cell membrane of distinct tissue resident cell populations or recruited from circulation. An imbalance in proteolytic activity of local cells involved in wound repair has been reported to result in persistent inflammation. Besides neutrophils, endothelial cells, fibroblast, and tissue macrophages release numerous proteases during wound healing. The endogenous enzymatic activity of proteases is regulated by endogenous inhibitors forming an intricate network. Alterations in the delicate balance of this network by the lack or altered activity of some agonists are associated with chronic inflammation (58, 59).

Uncoordinated production and deregulation of released proteases maintain the tissue damage, encouraging chronic inflammation and increasing cancer risk (60–62). Other factor promoting chronic inflammation is the uncoordinated production of the pro- and anti-inflammatory cytokines (63). Recent studies show that several cytokines exhibit a dual function according to their local concentration or interaction with other soluble factors (64). See **Table 1**.

**Table 1:** Key Cytokines and Growth Factors associated with acute and chronic inflammation.

Cytokine	Primary Target Cell	Biological Activity	Ref.
IFN-γ	Macrophages, NK, and T-cells	Increases MHC-I and-II molecules expression, neutrophil and monocyte function, and macrophage activation.	(65)
IL-1	NK and T-cells	Promotes systemic and local inflammation, fever, vasodilation, hematopoiesis, angiogenesis, leukocyte attraction, and extravasation; lymphocyte activation and acute phase response.	(66)
IL-2	T- and NK cells	Increases NK cells functions, Th0 cells activation, expansion, and differentiation into effector T-cells, controls immune response by maintaining Treg cells.	(67)
IL-4	T-cells, macrophages	Cytotoxic T-cells proliferation, enhances MHC Class-II molecules expression.	(68)
IL-6	T-, B-, and plasma cells	Regulation of acute phase response, activation of T helper cells. In combination with TGF-β induces Th17 cell differentiation during chronic inflammation.	(69–71)
IL-8	Neutrophils	Chemoattractant for neutrophils and T cells	(72)
IL-9	T- and mast cells	T-cell proliferation, mast cell growth, and Th2 cytokine secretion.	(73)
IL-10	T-, dendritic cells (DCs), and macrophages	Inhibits cytokine production by activated macrophages and DCs. Inhibits antigen-presenting capacity of monocytes and downregulates class-II MHC molecules expression.	(74)
IL-12	NK and T-cells	Promotes proliferation and cytotoxic effect of NK cells. Stimulates IFN-γ and TNF-α production in Th0 cells.	(75)
IL-17	Mucosal tissues, endothelial, T-, NK cells, and monocytes	Amplifies the inflammatory response induced by pre-existing tissue injury. Promotes production of some cytokines such as IL-8, MCP-1, CXCL1, G-CSF, GM-CSF, IL-1, IL-6, and TNF-α	(76, 77)
IL-18	Th1, NK, and B cells	Pro-inflammatory cytokine. Up-regulates cell adhesion molecules for leukocyte trafficking and induces nitric oxide synthesis. Cooperates with IL-12 inducing IFN-γ production from T helper and NK cells, leading to NK cell activation; up-regulates antigen presentation and exhibits antiviral and antitumoral functions	(78, 79)
IL-22	Fibroblasts, epithelial cells	Induces release of acute phase proteins	(80)
IL-23	T-cells	Stimulates the production of IFN-γ by Th1 cells, induces differentiation of CD4+ T-lymphocytes cells into Th17 and suppresses induction of Treg cells	(81)
IL-27	T-cells	**Pro-inflammatory** Induction of early Th1 differentiation. **Anti-inflammatory** Suppression of IL-2, IL-6, and IL-17. Reduces CD4+ T-lymphocytes differentiation into Th17, suppression of ROS production by macrophages and neutrophils. Inhibits DCs maturation.	(82–84)
TNF-α	Neutrophils, macrophages, monocytes, and endothelial cells	Immune regulation, fever, and inflammation	(85)
TGF-β	Different populations of immune cells	Enhances acquisition of myofibroblastic phenotype. Promotes and accelerates the wound healing process, acts as a chemoattractant for monocytes and fibroblast, potent inducer of the expression of α-smooth muscle actin, fibronectin, and collagen (major ECM proteins).	(86, 87)
GM-CSF	Myeloid-lineage derived cells	Promotes DCs differentiation in response to cytokine or inflammatory stimuli, activates the effector functions of myeloid cells at the resolution of inflammation to promote wound healing and tissue repair.	(88, 89)
G-CSF	Granulocytes	Stimulates proliferation and differentiation of granulocyte line, stimulates peripheral Th2-inducing DCs	(90)

In addition, immune and stroma cells are immersed in a varied collection of cytokines, growth factors, chemokines, and stroma factors in constant shift. Cells continuously exposed to these signals turn on or off numerous signaling pathways, impacting the phenotypic plasticity of distinct infiltrating immune cells. Chronic inflammation has been associated with the presence of Th17 cells that are differentiated from CD4+ Th1 lymphocytes. Differentiation to Th17 cells requires cytokines such as TGF-β and IL-6 or combination of IL-6, IL-1β, and IL-23 to activate the ROR-γ transcription factor. Differentiated Th17 cells secrete a wide variety of cytokines, including IL-17A, IL-17F, IL-21, IL-22, GM-CSF, IL-9, IL-10, and IFN-*γ*. Th17 cells are responsible for granulopoiesis and recruitment of neutrophils and macrophages in the injured zone; thus, they have been implicated in perpetuating chronic processes, such as psoriasis, Crohn’s disease, vasculitis, atherosclerosis, and asthma, among others (91).

During the acute inflammation resolution process, distinct immune cell populations are implicated. According to the lineage from which they were derived, immunoregulatory cells are classified into lymphoid-derived, Tregs, regulatory B (Bregs), and natural killer cells (NK cells); or myeloid derived, such as MDSCs, polymorphonuclear (PMN)- and monocytic (M)-MDSCs, regulatory macrophages, regulatory dendritic cells, regulatory neutrophils, and regulatory eosinophils. However, dysregulation in the migration, differentiation, and activity of these immune cells with immunoregulatory activity has been related with chronic inflammation. The activity and complete participation of these immunoregulatory cells in chronic inflammation are beyond the scope of the present manuscript. Excellent works have been conducted in this field (92–96).

**Figure 1:**
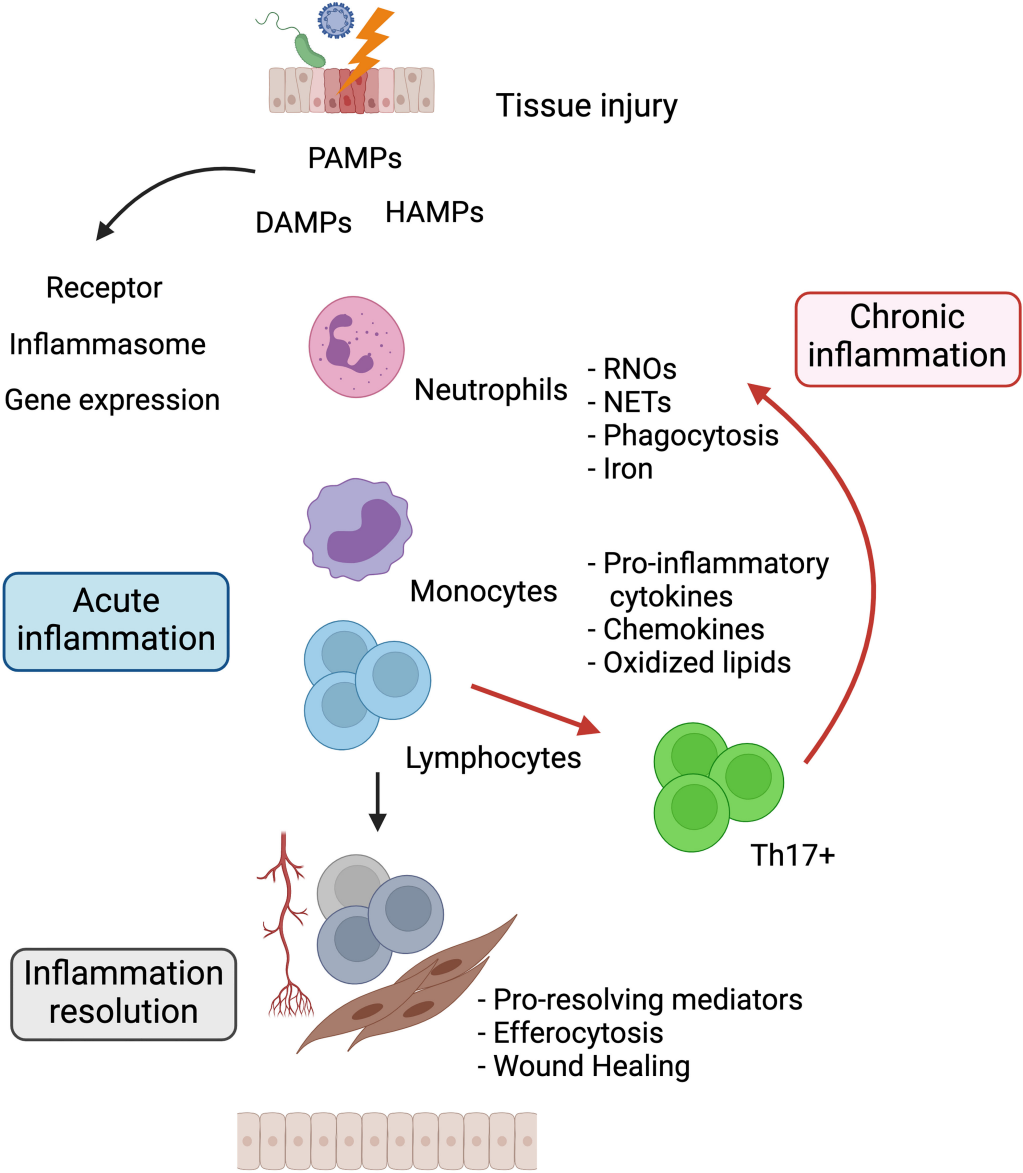
The relationship between acute and chronic inflammation. Harmful agents that damage tissues trigger the release of PAMPs, DAMPs, and HAMPs. Surrounding tissue cells sense these molecules through surface or intracellular receptors that trigger signaling pathways for the production of proinflammatory chemokines and cytokines. These soluble factors promote the migration of neutrophils and monocytes to the site of injury producing RNOs, NETs, increased iron, and phagocytizing noxious agents. Once the source of damage is eliminated, resolution of inflammation is induced, mediated by immunoregulatory cells and suppressor cytokines, restoring tissue homeostasis. Dysregulation and perpetuation of the aforementioned processes cause chronic inflammation. PAMPs, Pathogen-associated molecular patterns; DAMPs, Damage-associated molecular patterns; HAMPs, Homeostasis-altering molecular processes; RNOs, Reactive oxygen and nitrogen species; NETs, Neutrophil extracellular traps. Created with Biorender.com.

Despite the efforts made to understand and elucidate the molecular and cellular processes involved in chronic inflammation, the whole mechanisms that underpin the maintenance of this state remain unknown. In this setting, studies will help define biomolecules associated with the risk of chronic inflammation occurrence, especially its paradoxical effects in cancer development and progression. See **Figure 1**.

## Inflammation and Cancer

Dysregulated and unresolved chronic inflammation is recognized to play a major role in different types of pathologies as mentioned above. Special attention has been focused in the association of chronic inflammation with cancer development. The relationship between chronic inflammation and cancer is well known since the nineteenth century when Rudolph Virchow, based on his observations, proposed that cancer development was associated with the presence of immune infiltrate related to chronic inflammation (97). A century later, Dvorak reported that inflammation and cancer share common features such as proliferation, cell survival, induced angiogenesis, and migration (98). Nowadays, chronic inflammation has been considered as an enabling characteristic for tumor initiation and progression, helping to acquire additional cancer markers (99). Epidemiological studies suggest that 25% of cancer cases are associated to chronic inflammation (100, 101) and up to 15% of cancer malignancies are related to infectious diseases.

The typical oncogenic pathogen infections such as Epstein-Barr virus (EBV), human papillomavirus (HPV), hepatitis B virus (HBV), and hepatitis C virus (HCV) directly block the activity of tumor suppressor pathways, such as P53 and retinoblastoma (RB), disturbing the cell cycle.

The EBV infection has been linked to development of lymphocytic and epithelial malignancies.

Lymphoproliferative disorders such as Burkitt, Hodgkin, diffuse large B cell lymphomas, angioimmunoblastic T-cell, and extranodal NK/T-cell lymphoma, as well as gastric cancer and nasopharyngeal carcinoma of epithelial origin are some of the most frequent cases associated with this infection (102).

HPV is the most common sexually transmitted infection. High risk HPV types 16 and 18 are associated with more than 90% of cervical cancer, 85% of anal cancers, and 50% of penile, vulvar, and vaginal cancers. HBV infection causes acute and chronic hepatitis, and is a major risk factor for the development of hepatocellular carcinoma (HCC). HCV is another virus that shows tropism to the liver. Chronic HCV infection is associated with increased inflammatory cytokines and chemokines, and is the major risk factor for the development of HCC. This virus has been related to the development of some other carcinomas, as well as B-cell non-Hodgkin’s lymphoma. In general, oncogenesis by these infections can be mediated by i) oncogenic proteins encoded by virus genome, ii) oncogenic driver mutations that directly or indirectly induce chronic inflammation, iii) promote genomic instability that leads to carcinogenesis.

Excellent reviews of the molecular aspects and oncogenic pathways contributing to cellular transformation, concerning these and other viruses, have been previously published (103, 104).

With regard to bacterial agents, *Helicobacter pylori* infection has been recognized as a risk factor for gastric cancer. *H. pylori* induces inflammation by recruiting immune cells, which increase the production and release of RNOs to generate genomic instability causing the transformation of gastric epithelial cells (105). In this regard, some groups have found that *H. pylori* increases the activity of the Th17 lymphocyte subpopulation with consequent increase of IL-17 production, thus establishing an inflamed environment that stimulates the development and growth of cancer cells (106). However, this paradigm is shifting, as recent studies showed that *H. pylori* can directly interact with host genes that regulate the cell cycle, cell death, and other mechanisms of tumor suppression, thereby promoting the growth of incipient transformed cells (107).

*Fusobacterium nucleatum* upregulates host oncogenes through the interaction between its virulence factor FadA and E-cadherin expressed on epithelial cells. Clathrin-mediated endocytosis of the bacterium leads to phosphorylation of β-catenin and NF-κB activation, which upregulates inflammatory cytokine production. In addition, downstream activation of WNT, Myc, and Cyclin D1 promotes cellular proliferation to induce colorectal cancer (108, 109).

Enterotoxigenic *Bacteroides fragilis* (ETBF) is associated with risk to colorectal cancer (CRC) by recruiting Th17 cells and altering the STAT3/Th17 pathway (110). Excellent reviews about the participation of other pathogens in cancer development could be found elsewhere (107, 111).

With regard to the impact of sterile inflammation in the cancer development, environmental or lifestyle factors such as tobacco smoking, fine particle inhalation, and asbestos exposure are associated to lung cancer and mesothelioma (112). Wu et al. demonstrated that particulate matters present in air pollution cause autophagy-mediated inflammation in mice. Particulate matter activates TLR-4 that stimulates autophagy through mTOR inhibition. This event leads to activation of the NF-κB signaling pathway to upregulate the expression of proinflammatory cytokines, which might act as an important triggering event to develop cancer (113). Low-grade inflammation induced by excessive lipid accumulation, hyperglycemia associated with diabetes, and obesity increase the risk of different types of cancers, including liver, pancreatic, colon, breast, and other malignancies (114, 115). In addition, the presence of autoimmune diseases is now recognized as a risk factor of some types of cancer. For example, celiac disease, a systemic autoimmune disorder associated with chronic inflammation, is associated with the occurrence of gastric cancer and intestinal lymphomas in distinct cohorts of individuals (116).

**Figure 2:**
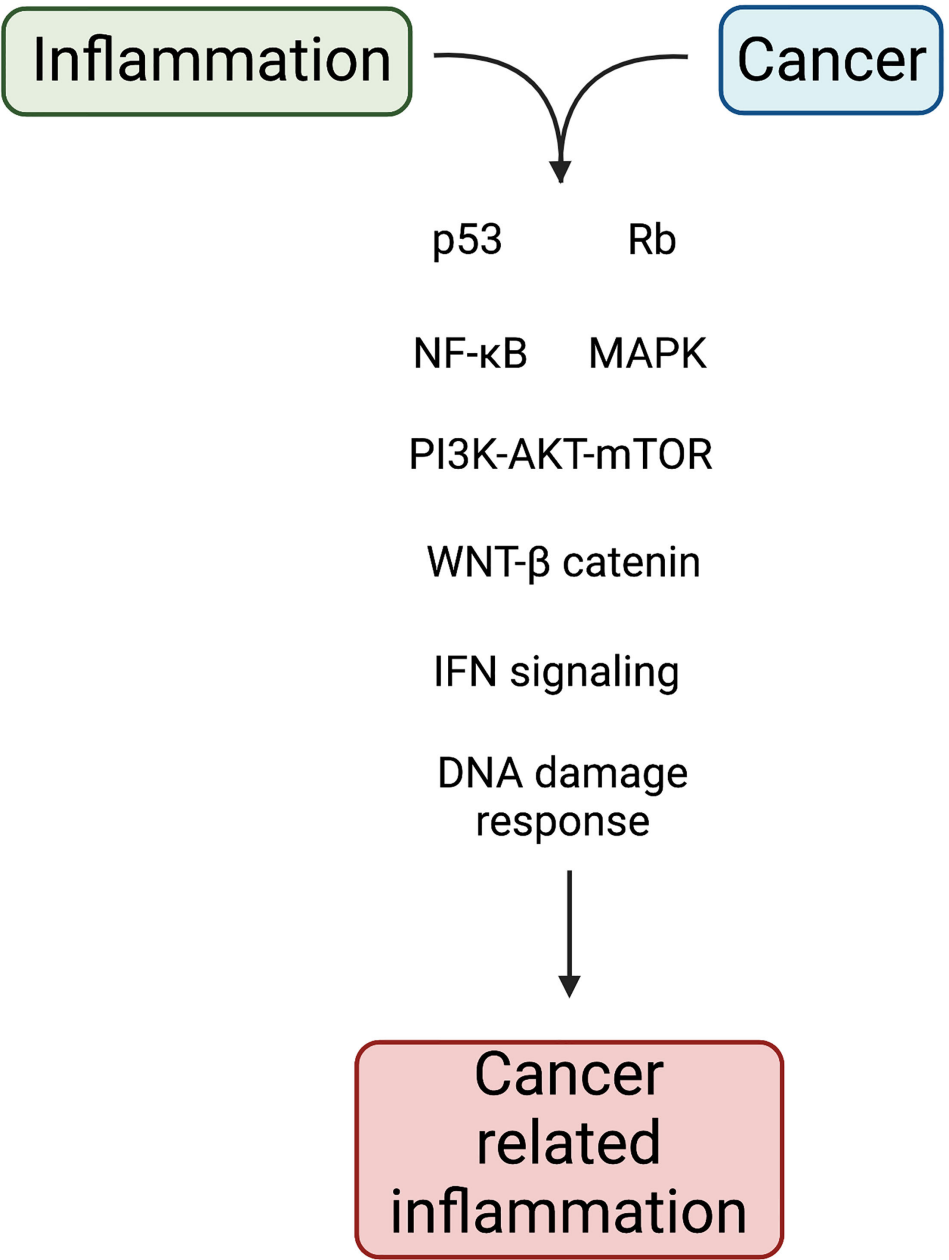
Molecular pathways linking inflammation and cancer. Chronic inflammation acts as an extrinsic pathway for cancer development. In tissue cells, genetic and epigenetic alterations act as intrinsic pathway to induce cell transformation. Both pathways impact on the activation of transcription factors that support inflammation and tumorigenesis. Created with Biorender.com.

As was mentioned before, phagocytic cells produce large amounts of iron. During chronic inflammation, iron participates in generating the oxidative burst required to kill phagocytosed pathogens. However, when deregulation in the production of iron occurs, ROS can cause protein denaturation, lipid peroxidation and DNA damage. As noted above, chronic inflammation caused by persistent pathogens can alter DNA and lead to tumor development. Likewise, this inflammation increases the genetic damage initially caused by physical, chemical or biological factors. Common signaling pathways between inflammation and cancer are depicted in **Figure 2**. In addition, recent findings have added a new avenue in the complex relationship between inflammation and cancer because oncogenic changes could induce a chronic inflammatory microenvironment.

Sustained proliferation of tumor cells requires a high demand for nutrients. Cancer cells utilize metabolic byproducts from immune and stromal cells to support cancer growth. Iron is one of these demanding nutrients and it has been reported that cancer cells capture several cytokines from the microenvironment to increase iron uptake and repress its efflux in order to maintain high levels of intracellular free iron (91, 117).

In addition, epithelial and immune stem cells carrying driver mutations or in early stages of cancer development enable the aberrant pathway signaling for blockage of cell death process and uncontrolled cell proliferation, causing tissue stress that favors a chronic inflammatory microenvironment. Gene-driver mutations in oncogenes such as Kirsten rat sarcoma (K-RAS), rearranged during transfection (RET), or MYC can continuously activate pathways that upregulate the expression and secretion of some proinflammatory cytokines, such as IL-1β, CSFs, IL-8, and CXC chemokines, among others (118–120). Continuous increase of inflammatory mediators and growth factors by stroma cells induces the inflammatory microenvironment that may contribute to the initiation and promotion of cancer.

The incipient and local inflammation leads to infiltration and increase in phagocytic cells that maintain a continuous release of RNOs, increasing mutations in oncogenes and tumor suppressor genes or epigenetic changes (121, 122). Mutagenic agents (e.g., peroxynitrite) may increase oncogenic transformations that cause non-synonymous mutations, which generate neopeptides in proteins that may act as tumor antigens. Moreover, metabolic alterations play as HAMPs to facilitate inflammasome activation and the synthesis and release of proinflammatory cytokines (123, 124). Dou et al. reported that cancer cells contain extranuclear chromatin (13). In varied cancers, cytoplasmic chromatin acts as a danger signal that activates the chromatin-cGAS-STING pathway, stimulating the expression of proinflammatory cytokines that, in a short term, activate the innate immune cells. However, persistent activation of this pathway leads to chronic inflammation induction and increases the genomic instability in tumor cells.

The evidence presented in this section suggests that perpetuated inflammatory response could facilitate the release of genotoxic agents, leading to a tumorigenic event. This process might be mediated by indirect or direct damaging of the genetic material of normal cells or through the established and preserved inflammatory microenvironment in which cytokines and growth factors stimulate the growth and development of nascent tumor cells. Together, these data demonstrate that sterile or non-sterile chronic inflammation may act as an extrinsic condition that precedes or promotes carcinogenesis. The crosstalk between tumor-inflammatory cells induces angiogenesis, facilitate metastasis, and modulate the antitumor immune response.

## Cancer Immunoediting Theory

Inflammation, as discussed above, is considered an enabling characteristic to promote tumor development. In this regard, inflammation might act as an extrinsic condition that transforms normal into tumor cells or could be an intrinsic event elicited by the aberrant activation of intracellular pathways due to mutations in driver genes. Stromal and immune cells participate in sustaining the elicited inflammatory state contributing in the acquisition of cancer biomarkers. However, in recent years, this view of the immune system as a driving force to promote tumorigenesis has been challenged by the understanding of the immune and stromal cell communication with cancer cells. Data obtained from *in vitro* studies and animal models show that specific genetic or molecular immune deletions exposed to genotoxic agents induce tumor development (125, 126). Schreiber’s group proposed the cancer immunoediting concept, explaining the tumor development and its progress in a host with a competent immune system (127). This theory is composed of three phases: the first involves the elimination phase, in which the immunosurveillance mediated by the innate cells, and also the adaptive immune response, help the total elimination of nascent tumor cells. This theory suggests that when tumor cells are not completely eliminated by the host immune response, a new phase known as equilibrium is induced. In this phase, the innate and adaptive immune cells continue to recognize and destroy susceptible immunogenic clones of the tumor that are continuously arising (128). This stage has been proposed as the longest in duration as tumor cells might enter in a dormant state induced by the immune response, a process called immune-mediated dormancy. In addition, other cellular events could be participating. Finally, in the escape phase, tumor cell clones become refractory to cytolytic molecules released by effector immune cells. Moreover, tumor cells affect the cytokine or growth factor microenvironment produced by the immune and stroma cells, impeding an efficient host immune response and thus causing the emergence of a clinically detectable tumor mass. At this moment, the immune and stroma cells in the tumor microenvironment switch from an antitumor to a protumoral activity contributing to the maintenance of the distinctive cancer biomarkers according to Hanahan and Weinberg (99).

### Interactions Between Innate and Adaptive Immune Cells and Nascent Tumor Cells

Early clinical oncology observations lead to discernment that neoplastic cells are recognized and eliminated by the host immune system.

A deeper knowledge of the nascent transformed cells and their subsequent neoplastic transformation for establishing a critical tumor-initiating cell has been achieved. However, the nature of critical interactions between nascent tumor and innate immune cells are still elusive due to obvious technical challenges related to *in vitro* and *in vivo* models. To overcome this obstacle, experimental models of chemotherapy-induced stress immunosurveillance have been developed to analyze the participation of innate immune cells (126). Based on these previous reports, we can highlight some aspects related to the nascent transformed cell, its ongoing transformation, and the early participation of the most important types of innate and adaptive immune cells. Knowledge of the distinct immune cell types and their roles in the antitumor immune response induction has led to the establishment of a tight collaboration between the innate and adaptive responses to control tumor progression.

Genotoxic agents are continuously impacting the genome of cells that constitute the human body and might promote the emergence of nascent transformed cells. From an immunologic perspective, the immunosurveillance theory suggests that distinct types of immune cells are continuously patrolling the body to detect and eliminate nascent tumor cells. For sensing, innate cells are armed with a collection of receptors for an immediate response against nascent transformed cells or their initial development. Immune cells with this capacity, particularly from the innate lymphoid cell (129, 130) compartment, have been identified, such as NK cells, γ-δ T-cells, and NKT, which perform the immunosurveillance.

NK cell activation is regulated by a strict balance between activation and inhibition signaling pathways controlled by their respective receptors (131–133). NK cells mediate the lysis of the target cells by releasing granzymes and perforin contained in their cytoplasmic granules. Release of these molecules at the zone of tight intercellular contact triggers target cell death (134). In addition to their main lytic function, some other NK subpopulations release chemokines and cytokines, with IFN-γ released earlier and as the most crucial cytokine (135).

Natural Killer T (NKT) cells were first detected in mice and some years later in humans (136, 137) and they have been incorporated as part of the innate immune response. NKT cells, unlike NK cells and T-lymphocytes, express a semi-invariant α-β T-cell receptor with restricted repertoire to recognize various endogenous and exogenous glycolipids or antigenic lipids associated to non-classical major histocompatibility complex (MHC)-like molecules, particularly the CD1d glycoprotein molecule (138–140). The α-galactosylceramide identified as a CD1d-restricted NKT cell antigen boosted the biologic importance of NKT cells in homeostasis and pathological conditions. Stimulation of NKT cells cause the immediate release of large amounts of cytokines and the same cytolytic machinery as NK cells (141).

γ-δ T-lymphocytes constitute a small proportion of T-lymphocytes infiltrating several tissues; therefore, they were formerly designed as intraepithelial lymphocytes. For their localization, these cells have a primordial participation in detecting tissue perturbation, infection, or tumors. γ-δ T-lymphocytes, as well as NK cells, express the NKG2D receptor that recognizes MICA/MICB and ULBPs proteins upregulated in stressed cells. The response called “lymphoid stress-surveillance” impedes the dissemination of infected or malignant cells. Subpopulations of γ-δ T-cells have been described to infiltrate distinct types of tumors, and some of them participate in secreting cytokines such as IFN-γ and TNF-α. Detailed information of this type of innate immune cell is indicated in Silva-Santos et al. (142).

In summary, NK, NKT, and γ-δ T-cells show effector activity mediated by the release of perforin and granzyme from cytoplasmic granules or mediate the cell death by the death receptor pathway. Moreover, NKT cells essentially release an array of cytokines for favoring activation of the cytotoxic activity of NK and γ-δ T-cells. Reports indicated that innate cells in addition to cytokine production also release chemokines to attract more immune cells.

A tight collaboration among NK, γ-δ T-cells, and NKT conforms a wide network to alert and react quickly to environmental changes for a successful destruction of the arising transformed cells. At this point, these cells participate in the immunosurveillance theory (143), which was incorporated as part of the elimination phase of the cancer immunoediting concept.

Based on the harmful agents inducing inflammation, PAMPs, DAMPs, and HAMPs in the microenvironment activate the endothelium because some of them show chemoattractant activity. In addition to cytokine production, innate cells also release chemokines. These soluble factors attract certain cell types as was previously mentioned in the acute phase of inflammation. In this initial and limited inflammation, neutrophils and mainly macrophages are the most abundant recruited cells to the injured tissue (144). Neutrophils and macrophages phagocytize dead cells and release RNOs causing a hostile oxidative damage that is mainly mediated by intracellular iron accumulation.

This oxidative stress generates cell death of susceptible viable tumor cells and simultaneously cause further genomic perturbations that increase genomic instability in residual viable cells. In this setting, the innate immune response is crucial to eliminate some susceptible tumor cells, while eliciting an antitumoral adaptive immune response.

When the transformed cells are not successfully eliminated by the innate cells, participation of the adaptive immune response is involved. In this step, conventional DCs, monocyte-derived DCs, and macrophages phagocytize transformed dead cells and process the altered self-proteins, harboring non-synonymous mutations, into small neopeptides. After, tissue DCs and macrophages migrate to lymphoid organs, where they mature. In the lymph node, mature DCs (mDCs) act as potent APCs. APCs present non-self-peptides in classes I and II MHC molecules to T-cells, which recognize the MHC-peptide complex through their T-cell receptor (first signal). In this interaction, expression of various costimulatory molecules is required (second signal), and simultaneously APCs release an array of inflammatory cytokines (IL-12, IL-23, IL-6, IL-27, IL-10, and TGF-β) (third signal). These signals are required for the adaptive immune response mediated by antigen-specific CD4+ T-lymphocytes clones. In a synchronized process in the lymph nodes, APCs also present tumor neopeptides to CD8+ T-lymphocytes through a process known as cross-priming.

In CD4+ T-cells after clonal expansion, they differentiate into effector Th1 lymphocytes that secrete an array of other cytokines (IL-2, IFN-γ, etc.) that provide autocrine and paracrine loops that continuously stimulate and expand tumor-specific CD4+ (Th1) and CD8+T-lymphocyte clones. During CD8+T-lymphocytes expansion and differentiation, gradually synthesize granzymes and perforin to become CTLs, which share the same cytolytic machinery with NK and NKT cells. Then, CD4+ and CD8+ lymphocytes with effector phenotypes migrate to reach the tumor zone and interact with malignant cells. Revisions that provided a detailed description of antigen processing and presentation to T-lymphocytes and also the array of involved cytokines have been previously published (145).

**Figure 3:**
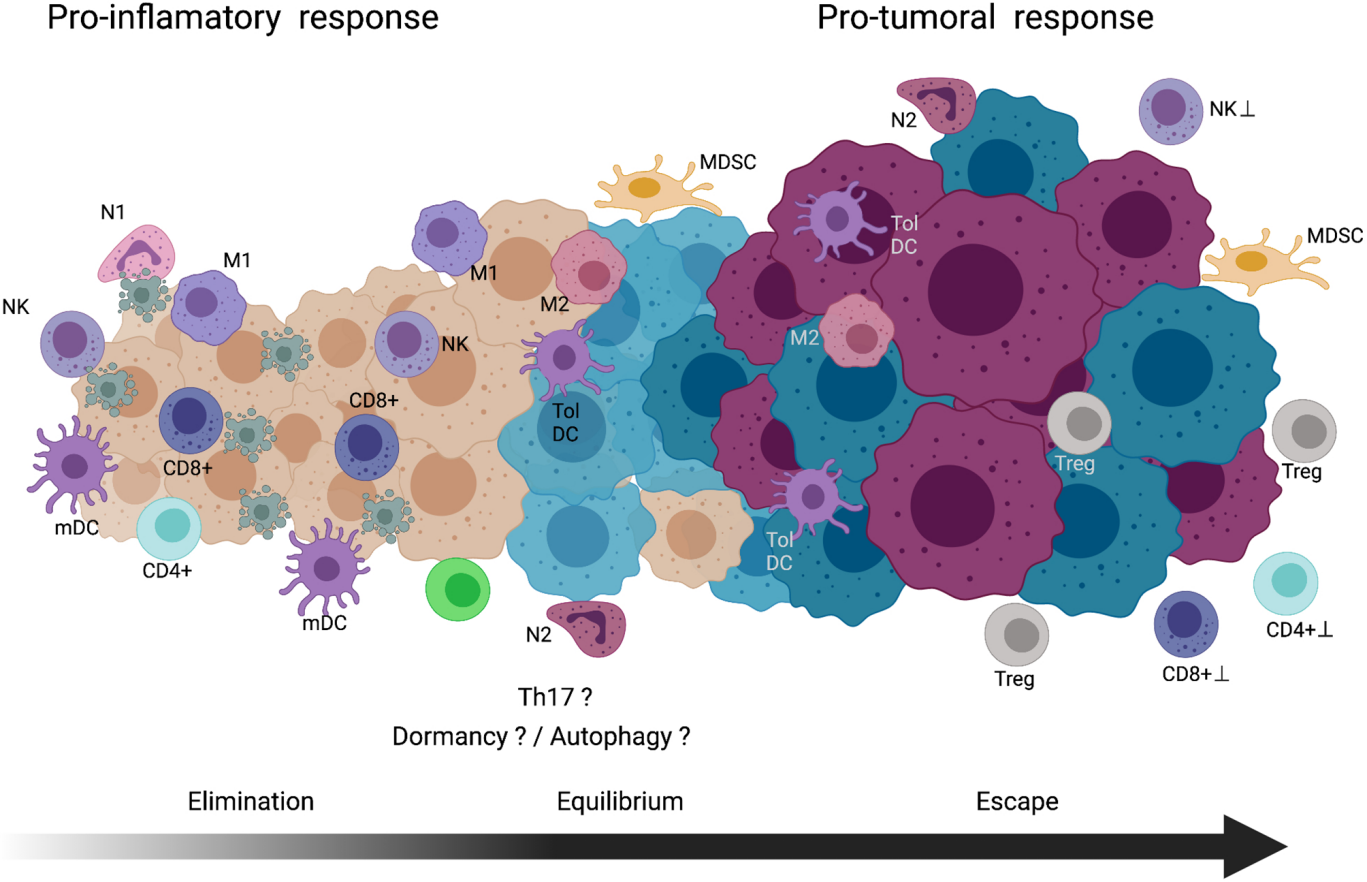
Cancer immunoediting theory. *Elimination phase*, immune cells with pro-inflammatory activity involved in the recognition and cell death of nascent tumor cells are indicated. *Equilibrium phase*, tumor zones composed of immune cells with proinflammatory and protumoral activites coexist in the tumor. In this long phase, dormancy and/or autophagy of the tumor cells might be occurring. Also, in the chronic inflammatory environment the Th17 cells could be participating. *Escape phase*, in this final step, tumor cells acquire mechanism to block the activity, cytocidal mechanisms of immune cells or maintain a microenvironment to promote in immune cells pro-tumoral activity. See text for further explanation. NK, natural killer cell; N1, type-1 neutrophil; mDC, mature dendritic cell; M1, type-1 macrophage; CD8+T, CD8 positive T-lymphocyte; CD4+T, CD4 positive T-lymphocyte; M2, type-2 macrophage; Tol DC, tolerogenic dendritic cell; N2, type-2 neutrophil; MDSC, myeloid-derived suppressor cell; Treg, regulatory T cell. Created with Biorender.com.

While CD4+ T- and CD8+ T-lymphocytes expand to effector cells, innate cells participate in destroying susceptible transformed cells in the tumor mass. Reports indicated that, in tumor bearing mice (146, 147), some cytokines (IL-2, IL-12, IL-15, and type I IFN) upregulates the NK cytolytic activity in collaboration with effector CTLs that in sufficient amount display an antitumor activity, resulting in the elimination of the initial tumor mass. See **Figure 3**.

Early and local tumor microenvironment is achieved in a coordinated and tightly regulated cellular inflammatory mechanisms that, after eliminating tumor cells, the resolution phase of the inflammation participates in the original tissue repair. When malignant cells were completely destroyed, most of the effector CD4+ T- and CTLs becomes tolerogenic or die by distinct mechanisms. However, a small proportion of the effector cells became memory T-lymphocytes, finishing the elimination phase (148).

### Equilibrium Phase

The immunoediting theory suggests that when the innate and adaptive immune responses do not eliminate all transformed cells and some of them maintain their viability, the remaining tumor cells will gradually increase its genomic instability. In addition, macrophages, neutrophils, tissue resident, or chemo-attracted phagocytic cells produce extrinsic factors such as RNOs that increase the genomic instability (149). Increased intrinsic alterations in the DNA repair machinery disrupted cell-cycle control and cell death are some of the affected processes.

As previously indicated, tumor recognition by NK and NKT is limited; therefore, their effector activity might be overwhelmed by transformed cells that are arising and gaining more and new mutations. The novel immunogenic antigens stimulate the participation of the adaptive immune cycle(s). Clones of CD4+ and CD8+ T-lymphocytes expressing TCR to recognize novel neopeptides are activated. This cycle repeats itself as long as immunogenic tumor neopeptides are generated and as a consequence of sustaining tumor cell proliferation, according to the cancer immunity cycle proposed by Chen et al. (150). In this scenario, during the equilibrium phase, the adaptive immune response helps eliminate the preceding and new clones of immunogenic tumor cells, and thus, an antitumoral activity is essential in this chronic inflammatory microenvironment.

In the equilibrium phase, tumor clones of the initial tumor might gain lethal mutations due to the high stochastic mutational rate that occurs as part of the cancer natural evolution (99). In this aspect, several mechanisms to eliminate genomically unstable cells have been reported, including the mitotic catastrophe (151, 152). Cell death for this condition supports a chronic inflammatory environment in which the antitumoral activity of immune cells should be preponderant. However, some cytokines and growth factors (IL-17, IL-6, IL-10, TGF-β, GM-CSF, etc.) are simultaneously released in the microenvironment; hence, the proliferation of malignant epithelial cells containing the genetic arsenal can stimulate tumorigenesis (153). These tumor clones gradually acquire growth advantage by forming a critical tumor mass that allows them to resist the effect of cytotoxic molecules released by the immune cells or induce microenvironments that change the phenotype of immune cells into a protumoral activity.

The equilibrium phase is considered as the step with longest duration. An event that could preserve this long duration is tumor dormancy, a mechanism characterized by both inhibited proliferation and cell death. Several studies provide data that the aberrant organization of tumor growth leads to loss of tissue architecture, inducing a deficient crosstalk with the extracellular matrix components. Loss of this communication supports tumor dormancy (154). Moreover, gradual increase in tumor mass could establish an oxygen- and nutrient-limited environment, due to the absence of factors involved in neovascularization turn on, resulting in a stage of cell dormancy. This event is reversible when the angiogenic program is activated.

In addition, as innate and adaptive immune systems destroy proliferating tumor cells, some malignant cells may enter into cellular arrest reducing their proliferation and keeping them clinically dormant. Other possible mechanisms may be involved in tumor cell dormancy is the histologic type of cancer. Undoubtedly, a deeper knowledge of these phenomena during the equilibrium phase should generate new markers and therapeutic targets related to earlier cancer stages.

Autophagy is another even that could be associated with the equilibrium phase and caused by nutrient deficiency in tumor microenvironment. The role of autophagy in cancer has been considered as dichotomic, which might act as a tumor suppressor mechanism during early tumorigenesis but might stimulate the growth and survival of tumor cells in advanced stages (155, 156). Some reports indicate that dormant cells upregulate autophagy in order to meet the metabolic demands to sustain their viability (157). Interestingly, several studies reported that tumor cells increase the autophagy rate to evade the NK, NKT, γ-δ T-cell, and CD8+ T-cell activities (158, 159). Baginska et al. revealed that in MCF-7 cells, autophagy impairs the cytotoxic activity of NK cells by sequestering and degrading the granzyme B inside the autophagosomes under hypoxic conditions. In addition, Yamamoto et al. recently found that, in a mouse model of pancreatic cancer, tumor cells increase autophagy to selectively degrade class I MHC molecules, thereby reducing the expression of neopeptides and their subsequent recognition by CD8+ T-lymphocytes (159, 160). Overall, these findings suggest that during the equilibrium phase, tumor dormant cells could upregulate autophagy that sustains cell viability and hinders the cytotoxic effect of innate and adaptive immune cells and thereby helps in tumor sculpting. See **Figure 3**.

To sum up, in the equilibrium phase tumor development will depend on diverse factors such as the type of agent involved in the tumor induction, oncogenic signaling pathways implicated in the cellular events in the tumor, histological type of cancer, microenvironment in which the primary tumor is induced, genetic susceptibility of the patient, and some several other factors such as gender and age, among others.

### Escape Phase

This is the latter stage of cancer immunoediting. In this step, the accumulation of genomic alterations, conferred by gradual or catastrophic events along different stages of tumor development, originate primary tumors with high intratumoral heterogeneity (161). These clones have undergone a long selection process, rather due intrinsic mechanisms aimed to eliminate cells with aberrant, genetic alterations and to the pressure exerted by the host immune systems, which eliminated the immunogenic tumor clones that sculpt the tumor phenotype.

The spatiotemporal interplay of oncogenic driver characteristics of the tumor cell, its interaction with various immune and stroma cells, and matrix elements impact in the establishment of inherent complex and shifting microenvironments with distinct biological variability. These microenvironments allow generation of distinctive microhabitats that over time lead to development of diverse cellular niches, which have been reported on the same surgical specimen (162). In this step, tumors establish an immunosuppressive environment to evade the recognition and destruction of the host immune response. Diverse compounds of the tumor microenvironment derived from metabolic changes, reduced oxygen supply, altered tissue architecture and other factors encourage the release of growth factors, cytokines, and soluble ligands that reduce the tumor antigen recognition, block the immune cell activation or inhibit the effector phase of the cytolytic immune cells. Tumor cells secrete numerous chemokines and cytokines with protumoral activity, such as TGF-β, IL-10, IL-4, IL-6, G-CSF, and GM-CSF, among others. Local overproduction of these cytokines by tumor cells, in addition to that produced by immune and stromal cells, maintain an altered environment for tumor progression. In particular, cytokines such as TGF-β, IL-6, and IL-10, alone or in combination, can promote the expression of immune cells with regulatory function, such as Tregs, MSDCs, M2 macrophages, N2 neutrophils, and immature DCs (iDCs).

Another point of complexity is the tumor and immune cellular heterogeneity. The proportions, spatial distribution, and functionality of the cells infiltrating the tumor to produce cytokines and growth factors vary in each tumor even those with the same histological type (intertumoral heterogeneity). Moreover, these characteristics have been demonstrated to vary in different specimen zones, thereby supporting the notion of intratumoral heterogeneity. The primary immune cell identified in tumor infiltration, due to its importance in tumor cell elimination, is the CD8+ T-lymphocytes (CTLs). In this immune population, proteins related to the cytotoxic potential such as granzyme B and perforin have been analyzed. Furthermore, expression of checkpoint markers such as CTLA-4 and PD-1 have been associated with T-cells undergoing chronic stimulation, a phenomenon known as exhaustion (163). Various studies also reported that due to cell plasticity in response to environmental cytokines, high infiltration of Treg cells, M2 macrophages, DCs, or MDSCs can be detected (164, 165).

Another event that has been reported to affect the tumor cell heterogeneity is the epithelial-mesenchymal transition (EMT), triggered by inflammatory stromal cells and ECM components. During the tumor growth, some zones of the tumor mass reach a size that prevents oxygenation, inducing hypoxic zones and production of cells with more aggressive phenotypes. TGF-β, a key player for the EMT, is produced by neutrophils, platelets, M2 macrophages, MDSCs, and tumor cells themselves. Furthermore, TGF-β is released from its latent form from ECM proteins (166, 167). The EMT signature has been associated with expression of different immune checkpoints inhibiting the effector cells (168–170). Despite the significant progress in this issue, more studies are required to understand the complex and dynamic circuits associated with the EMT transition, the gradual acquisition of protumoral phenotypes by immune cells, and the resistance to cancer therapies, mainly immunotherapy.

Another mechanism participating in the escape phase of immunoediting theory is the production of immunosuppressor metabolites. T-lymphocytes have been demonstrated as the principal cells producing IFN-γ to maintain a chronic inflammatory environment. However, as a negative feedback regulatory mechanism, they also induce the expression of indoleamine 2,3-dioxygenase 1 (IDO-1) in endothelial cells and stromal fibroblast. IDO-1 degrades tryptophan, an essential amino acid for lymphocyte survival. The regulatory environment created by this mediator can offer a pathway of tumor immune escape. Numerous reports indicated that IFN-γ induces IDO-1 expression in tumor cells and is associated with a negative prognostic factor in several cancers (171). In tumor microenvironment, IDO-1 produced by cancer cells, DCs and plasmacytoid dendritic cells (pDCS), induces downregulation of the ζ chain of the TCR in T-lymphocytes. In CD8+ T, γ-δ T-cells and NK cells decrease the expression of degranulation marker CD107a and granzyme B. Moreover, IDO-1 acts as a potent suppressor of CD8+ T-lymphocyte activation, stimulates the differentiation of naïve CD4+ T-cells into FoxP3+ Tregs, promotes T-lymphocyte cell death, and has been reported to correlate with the expression of PD-1 and PD-L1 immunologic checkpoints. All these evidences, using animal models and human studies, demonstrated the biological importance of IDO-1 in promoting and facilitating tumor progression (172).

Chronic inflammation is maintained during tumor development. In early stages of tumor development (elimination phase), the inflammatory response exerts an antitumoral effect. However, in advanced stages of cancer, deregulation or overproduction of chronic inflammation mediators show protumoral activity by inhibiting the host immune response. The essential amino acid L-arginine (L-arg) participates in the immune cell proliferation. During the repair phase of acute inflammation, macrophages recruited to the injured area express arginase, an enzyme that hydrolyzes L-arg to L-ornithine, which is then degraded to proline for collagen synthesis (173) or forms polyamines that stimulate cell proliferation (98). Reports indicated that tumors can produce L-arginase; however, most studies found that the production of L-arginase is derived from tumor-associated stroma cells, including macrophages, DCs, granulocytes, monocytes, and mast cells, grouped as MDSCs. Distinct local factors from DAMPs to varied environmental conditions such as hypoxia, nutrient deficiency, cellular metabolites, products derived from the ECM, growth factors, and cytokines stimulate the arginase production in MDSCs. In tumor microenvironment, starvation of L-arg by MSDCs downregulated the CD3 ζ chain of the TCR in lymphocytes; reduced the MHC molecule expression hampering the tumor antigen presentation; restricted viability, proliferation, and effector activity of the NK cells; and induced the presence of alternative macrophages M2 and N2 neutrophils. All and other alterations promote the protumoral activity of the immune response. An excellent review of MSDCs induction and their importance in tumor microenvironment has been recently published by Grzywa et al. (174). See **Figure 3**.

Under physiological conditions, the immune cell response is strictly regulated by a balance of stimulatory and inhibitory signals to maintain self-tolerance or by minimizing the duration and extension of inflammation. Receptors and ligands, both members of this attenuating pathway, have been designed as “immune checkpoints.” The roster of this type of molecules is rapidly expanding, including but not limited to the following: CTLA-4, PD-1, LAG-3, TIM-3, TIGIT, GITR, and CD96 Also, some members of the B7-CD28 family [B7-H3, V-domain Ig suppressor of T-cell activation (VISTA), and B7-H7], Siglec-7 and Siglec-9, CD200, CD47, and recently HLA-G have been reported.

Several authors have reported that TILs express distinct checkpoints and have been associated with immune response inhibition. In addition, reports indicate that some cancers upregulate the expression of some checkpoints or corresponding ligands. During cancer development, cancer-driving gene alterations and microenvironmental factors have a key role on the ligands or checkpoint molecular expression on cancer cells (175).

The VISTA is a recently discovered immune checkpoint. In human cancers, VISTA expression has been reported in melanoma, hepatocellular carcinoma, colorectal, oral squamous cell carcinoma, gastric carcinoma, acute myeloid leukemia, ovarian cancer, and non-small cell lung cancer (NSCLC) (176, 177). On ovarian cancer cells, VISTA expression is associated with suppression of T-cell proliferation, infiltration, and cytokine production (178). However, in melanoma, VISTA has been reported to promote the induction and maintenance of Treg cells (179). Wang et al. identified that V-Set and immunoglobulin domain containing 3 (VSIG-3) molecule is a putative ligand of VISTA. In this regard, VISTA/VSIG-3 interaction inhibits proliferation of T-cells and diminish the production and release of some chemokines and cytokines such as IFN-γ, IL-2, IL-17, CCL5/RANTES, CCL3/MIP-1 α, among others (180). It has been demonstrated that VSIG-3 is over-expressed in colorectal and intestinal cancers, as well as hepatocellular carcinomas (181).

Galectins are a family of proteins that bind to a specific glycan. In cancer cells, aberrant glycosylation of these proteins has been reported. Secreted galectin-9 facilitates immune suppression by killing CTLs and impairing the NK cell activity. In contrast, the more likely detected membrane expression of galectin-9 protects tumor cells against CTLs-induced death. Yasinska et al. recently reported that cancer cell lines from the brain, colorectal, kidney, blood/mast cell, liver, prostate, lung, and skin expressed detectable amounts of both TIM-3 and galectin-9 proteins (182).

In addition to APCs and Treg cells in the tumor microenvironment, cancer cells express CD155 (PVR) and CD112 (PVRL2, nectin-2) molecules, which are ligands of the T-cell immunoreceptor with immunoglobulin and ITIM domain (TIGIT), DNAM-1 (CD226), TACTILE (CD96), and the recently described PVRIG checkpoint. TIGIT, expressed in activated CD4+ T- and CD8+ T-lymphocytes and NK cells, binds to CD155 or CD112 ligands, triggering a signaling pathway that blocks effector T-lymphocyte functionality, thereby acting as an important tumor evasion mechanism (183, 184).

The member of the B7 superfamily of immune modulatory ligands B7-H3 (CD276) is an additional checkpoint related to B7-H1 (PD-L1), B7-DC (PD-L2), B7-H2 (ICOS-L), and CTLA-4 ligands B7-1/B7-2 (CD80/CD86). Normal tissues express B7-H3 and are highly overexpressed in numerous carcinomas. In most cases, B7-H3 expression is associated with poor outcomes in melanoma, leukemia, prostate, colorectal, and ovarian cancers (185–191). In cancer cells, B7-H3 has been associated with the promotion of protumorigenic functions, such as angiogenesis, migration and invasion, EMT, metabolism, and chemoresistance (189).

PD-L1 is by far one of the most important and studied ligands of checkpoint molecules in cancer cells since its expression has been employed as a prognostic marker. To this respect, PD-L1 is expressed in renal cell carcinoma, NSCLC, colorectal, breast, gastric, papillary thyroid, and testicular cancers (192). Recently, Hou et al. reported that phosphorylated STAT3 is associated with PD-L1 in the tumor cell cytoplasm in hypoxic conditions, the binding that facilitates nuclear import of PD-L1. Authors describe that in multiple cancer cell types, including lung, breast, liver, and ovarian cancers and melanoma, nuclear PD-L1 facilitated TNF-α-induced apoptosis by enabling tumor cell necrosis (193). Perhaps in cancer patients with favorable clinical response to the anti-PD-1 or PD-L1 antibody-based therapy and with biopsies containing a high infiltration of T-lymphocytes, particularly effector CD8+ T-cells, in the intraepithelial compartment or intraepithelial and stroma zones that are also PD-1+ or PD-L1+, an analogous phenomenon described by Hou may be occurring. Immunotherapy-mediated brake release, in addition to DAMP shedding by tumor necrosis, might trigger an efficient activation of the host immune response. Further studies assessing variations in different subsets of circulating immune cells throughout the checkpoint therapy are required to analyze these issues.

**Figure 4:**
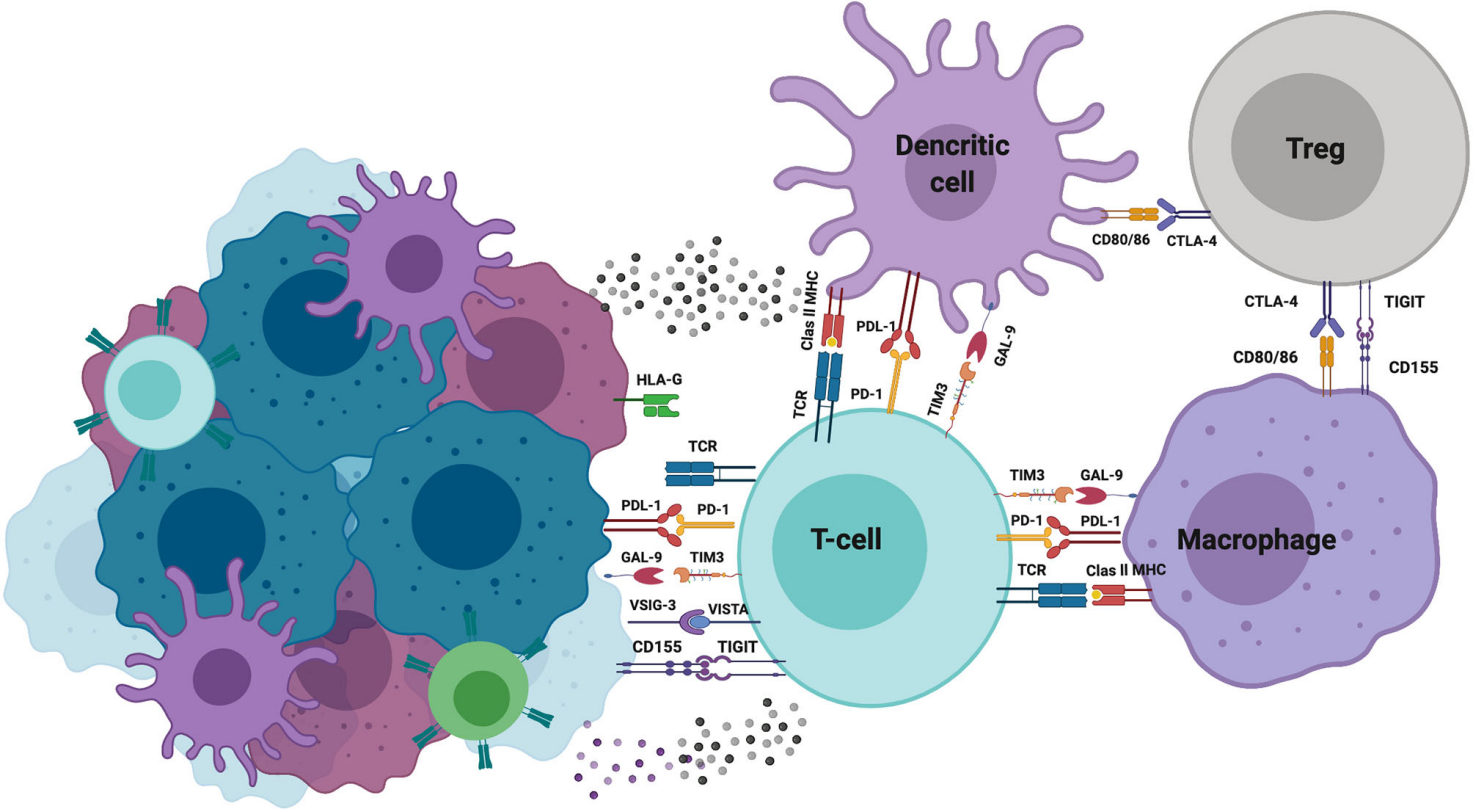
Immune checkpoint molecules. Schematic representation of the main checkpoint molecules with inhibitory activity expressed in immune and tumor cells as a mechanism of immune evasion. Created with Biorender.com.

Many studies have reported that advanced stages of cancer exhibit impaired expression of class I MHC molecules caused by mutations or loss heterozygosity of genes involved in the machinery for tumor antigen presentation (194). However, in pancreatic ductal adenocarcinoma, MHC-I molecules are localized into autophagosomes and lysosomes for selective degradation (160). The reduced MHC-I molecule expression at the cell surface as consequence of any alteration is a tumor evasion mechanism that impedes the interaction of effector CD8+ T-lymphocytes for specific tumor destruction. In contrast to the alteration of class I MHC expression, several studies have showed that many tumors upregulated the MHC-like HLA-G molecule, possibly due to deregulated post-transcriptional mechanisms. In addition to HLA-G membrane expression, tumors can transfer part of their membrane HLA-G to immune cells or release this molecule in exosomes. Increased HLA-G in the tumor microenvironment induces remarkable inhibition of the host immune response, which can be considered as an important immunoregulatory molecule in cancer (195–197). However, further studies are necessary to determine the biological importance of the overproduction of the HLA-G molecules in tumors. See **Figure 4**.

Undoubtedly, new molecules exhibiting immune checkpoint activity will be identified on the tumor microenvironment. Understanding the effects that they orchestrate through the signaling pathways that are activated in both immune cell infiltration and tumor cells is required. These emerging molecules combined with those already described will be useful markers that strictly determines the prognosis of cancer patients.

## Anti-Inflammatory Drugs in Cancer Treatment

As was previously discussed, inflammation is strongly linked with cancer development. In this context, key inflammatory mediators such as cytokines, growth and transcription factors, and signal transducers promote some of the hallmarks of cancer i.e., sustained growth, cell death evasion, genomic instability, inhibition of immune-mediated destruction, angiogenesis, and the activation of migration-invasion programs (99). For this reason, the use of anti-inflammatory drugs represents a promising therapeutic strategy for prevention and cancer treatment.

In recent years, several pre-clinical and clinical studies have demonstrated that anti-inflammatory drugs, either alone or in combination with anti-tumoral agents, could promote tumor cell destruction (198–200). For example, cyclooxygenase type 2 inhibitors such as celecoxib, have demonstrated to promote cell death by interfering with the mitochondrial transmembrane potential in an *in vitro* model of mouse hepatoma (201). A similar effect was found by Jeon et. al, in human breast cancer cell lines MCF-7 and MDA-MB-231 which were susceptible to cell death after exposure with celecoxib and luteolin (202). In support of this, FDA approved the use of this drug as an adjuvant for the treatment of familial adenomatous polyposis for preventing the development of colorectal cancer (203).

Additionally, anti-inflammatory drugs have demonstrated to enhance the effect of conventional anti-tumoral drugs decreasing their toxicity by interfering with their pharmacokinetics and metabolism. Administration of glucocorticoids previous to treatment with gemcitabine or carboplatin promoted the accumulation of these drugs at the tumor site in a mouse xenograft model (204). However, no significant differences in plasmatic concentration of docetaxel were found when combined with prednisone in prostate cancer patients (205). For this reason, more studies enrolling an increased number of patients are required.

Given the examples mentioned above, in recent years, the discovery and the pre-clinical evaluation of novel anti-inflammatory agents as anti-tumoral drugs have been performed. In this setting, pre-clinical studies have shown that agents based on COX-2 inhibitors or chemically modified NSAIDs are able to inhibit cancer growth by targeting common inflammation-cancer pathways such as MAPK, PI3K/AKT, STAT3, and NF-κB (199). Special attention has been focused in the activity of chemical compounds derived from natural products which, beside of show anti-inflammatory properties, have demonstrated anti-tumoral activity. For example, derivates from *Larrea divaricate* or *Artemisa rubris* plants are potent 5-lypo-oxygenase (5-LOH) inhibitors (206, 207). In this regard, 5-LOH inhibitors such as embelin have shown a potent anti-tumoral activity by inducing cell death, cell cycle arrest, and inhibition of secretion of pro-inflammatory cytokines, growth factors, and metalloproteinases (208–210).

This evidence supports the notion that targeting inflammation is a strategy to prevent and treat several types of cancer. However, extensive research is needed in order to understand the molecular mechanisms by which the anti-inflammatory agents could interfere with biological processes related with cancer cells. Additionally, it is important to consider that the activity of the anti-inflammatory agents might also impact/affect other cells present in the tumor microenvironment, mainly immune cells. For this reason, more studies incorporating *in vivo* models are required for elucidating the underlying activity of current and novel anti-inflammatory drugs in tumor microenvironment.

### Concluding Remarks and Perspectives

Acute inflammation is a dynamic, synchronized, and highly regulated process in response to an external infectious or non-infectious insult culminating with injured tissue healing. In this review, we summarized advances on the mechanisms controlling the inflammatory process. Meanwhile, pathogens release metabolism-derived or degradation products, known as PAMPs, and damaged cells release DAMPs. Currently, some lipophilic metabolites, termed as HAMPs, are emerging as important players in triggering inflammation since their aberrant production is recognized as a danger signal. The importance of PAMPs or DAMPs and HAMPs at the initial steps of inflammation was highlighted. Cells and molecules, including HAMPs, involved in the inflammation resolution were also indicated. Although significant advances have been made in several aspects of inflammation development and resolution, further research is required to discern the signaling pathways and gene expression regulation in controlling and regulating tissue repair. Undoubtedly, knowledge of these aspects will lead to the development of new treatments to prevent the progression of a chronic process and improve wound healing.

However, when inflammation persists and tissue homeostasis is lost, a chronic process is triggered. The role of inflammatory cells and overproduction of biomolecules contributing to this phenomenon were mentioned. Continuous production and release of PAMPs, DAMPs, and HAMPs promote incessant arrival of inflammatory cells that damage the normal tissue by releasing proteases and oxidant agents. Loss of some molecules or failure in recruitment of immune cells with regulatory activity, as antagonists of stimulatory signal exacerbation, was indicated. In contrast, participation of Th17 immune cells in perpetuating the chronic process was suggested. Chronic dysregulated and unresolved inflammation has been associated with the risk of cancer development and has been considered as a tumor-enabling characteristic. Although the relationship between inflammation and cancer is well established since the last centuries, this classical paradigm of chronic inflammation, maintained by some types of pathogens, as the cause of cancer is changing. With this point, several groups have reported the activation of driver genes in the host cells by certain pathogens. In addition, oncogenic changes in transformed cells have been implicated in upregulating the expression and secretion of chemokines and cytokines for maintaining an inflammatory microenvironment. This environment facilitates the process of tumorigenesis by increasing genomic instability and promoting proliferation.

As tumor develops in a host with a competent immune system, the cancer immunoediting concept suggests that innate and adaptive immune responses, triggered in a regulated inflammatory environment, recognize and eliminate nascent tumor cells or tumor in earlier stages.

Due to genomic instability, a gradual gain of genetic and epigenetic alterations leads to the emergence of distinct tumor cell clones becoming refractory to the recognition and elimination mechanisms orchestrated by immune cells. During this process, tumor cells initiate programs to avoid proliferation by the remaining dormant or upregulate autophagy for sustaining cell viability and hindering the cytocidal effect of the immune cells. This large equilibrium stage overlaps with the evasion phase, in which tumor cells create an environment that modifies the phenotype of immune cells to promote tumor growth. In addition, different mechanisms that impede tumor recognition by immune cells were described as another approach of tumor evasion in this review. Studies in biopsies of cancer patients exhibit a heterogenous distribution of distinct subsets of tumor-infiltrating immune cells. Significant progress has been made related to the molecular expression with inhibitory potential known as immune checkpoints. These data have been associated with clinical outcomes of cancer patients and resulted in the development of immunotherapies against checkpoint molecules. However, not all patients are benefited from immunotherapy, even when they exhibit a similar immunophenotype or proportions of immune cells infiltrating the tumor; thus, different factors may be involved in the failed response. One of these factors may be associated with the novel expression of checkpoint in tumor cells, besides the ligand. Understanding the effects, they orchestrated through the signaling pathway that activate tumor cells is required. A rigorous understanding of the progression and complexity of the interactions leading to overexpression of immune checkpoint array in immune and tumor cells environment will overcome the resistance mechanisms to this type of immunotherapy.

Despite great advances in understanding the relationship of the inflammatory response in the development and progression of cancer, knowledge on critical aspects involved in this process will impact in the development of forthcoming therapies for controlling cancer growth and increasing patient survival. Though the *in vivo* models have allowed to gain depth in the knowledge with respect of the anti-tumoral activity of anti-inflammatory agents, not always the results obtained from these models could be translated to cancer patients. Undoubtedly, the human intellect will achieve a better understanding of these phenomena by developing more complex and dynamic models for studying the relationship among the immune cells, cancer progression, and the effect of anti-inflammatory agents.

## Author Contributions

DA-C, RC-D and MP-M organized the entire manuscript, wrote the draft and revised the last version of the manuscript. DA-C and MG-V wrote the acute inflammation section. RC-D and MP-M wrote the chronic inflammation section. DA-C, LI-V, RC-D, and JL-G wrote the inflammation and cancer section and cancer immunoediting theory section. MM-F and AC wrote the tumor evasion mechanisms section. RC-D and JL-G wrote the anti-inflammatory drugs section. **Figures 1**–**4** were designed by RC-D, DA-C, MP-M, and JL-G. **Table 1** was designed by JL-G and MP-M. All authors contributed to the article and approved the submitted version.

## Funding

The manuscript was partially funded by Consejo Nacional de Ciencia y Tecnologia (CONACYT) (grant number: 284775).

## Conflict of Interest

The authors declare that the research was conducted in the absence of any commercial or financial relationships that could be construed as a potential conflict of interest.

## Publisher’s Note

All claims expressed in this article are solely those of the authors and do not necessarily represent those of their affiliated organizations, or those of the publisher, the editors and the reviewers. Any product that may be evaluated in this article, or claim that may be made by its manufacturer, is not guaranteed or endorsed by the publisher.
